# Novel Therapeutic Targets for Tumor Microenvironment in Cancer

**DOI:** 10.3390/ijms24087240

**Published:** 2023-04-14

**Authors:** Roberto Bei, Laura Masuelli

**Affiliations:** 1 Department of Clinical Sciences and Translational Medicine, University of Rome “Tor Vergata”, 00133 Rome, Italy; bei@med.uniroma2.it; 2 Department of Experimental Medicine, University of Rome “Sapienza”, 00161 Rome, Italy

The various immune effector cells that infiltrate the tumor microenvironment (TME) play a key role in directing the outcome of tumor growth [1,2,3,4,5,6,7]. The immune response plays a dual role in the TME by eradicating cancer cells or supporting tumor growth. Cells of the innate immune system and cytotoxic/helper T lymphocytes, which produce interferon-γ (IFN-γ), are engaged in the TME and induce cell death, the additional activation of natural killer (NK) cells and macrophages, and stimulation of the humoral response and inflammation [1,2,3,4,5,6,7]. On the other hand, the occurrence of different subsets of T cells is dependent on the patterns of cytokines and on receptor expression [1,2,3,4,5,6,7]. Cytotoxic CD8+ T cells (CTLs) are the most powerful effectors involved in the anticancer immune response [7]. TH1-polarized CD4+ T cells provide help for CTLs, produce effector cytokines that exhibit antitumor activity, and induce humoral responses against tumor antigens [4,8], while TH2-polarized CD4+ T cells induce tumor-associated macrophages (TAMs) to secrete pro-tumoral survival factors and cytokines [4,8]. In addition to TH2 CD4+ T cells, several mechanisms participate in cancer immunoevasion and immunosuppression. Tumors may counteract the immune system by employing immunosuppressive leukocytes, fibroblasts, and soluble factors [9]. Regulatory T cells (Tregs) are able to suppress immune responses by impairing the activity of dendritic (DC) and NK cells and by releasing immunosuppressive cytokines or other metabolites [10]. Fibroblasts that are present in the TME, and thus, defined as cancer-associated fibroblasts (CAFs), release cytokines and chemokines, as well as extracellular matrix (ECM) molecules that are able to support a pro-tumoral microenvironment [11]. The persistence of proinflammatory mediators contributes to cellular transformation and progression [1,2,3,4,5,6,7]. This Special Issue of the International Journal of Molecular Sciences, entitled “Novel Therapeutic Targets for Tumor Microenvironment in Cancer”, comprises four original research papers and one review article. These papers highlight current research that further elucidates the mechanisms that support the development of the pro-tumoral microenvironment and the growth of cancer cells, and suggest tools to counteract it.

Jacenik et al. identified mitogen-activated protein kinase-activated protein kinase 2 (MK2) as a novel regulator of pancreatic neuroendocrine tumor (PNET) progression. MK2 showed a pro-tumorigenic role in a RipTag2 transgenic murine model of PNETs. Indeed, the inhibition of MK2 ameliorated mouse survival and prevented PNET progression by suppressing pro-tumorigenic macrophage-related cytokine/chemokine production (granulocyte colony-stimulating factor, interleukin (IL)-6, IL-10, keratinocyte chemoattractant, leukemia inhibitory factor, monocyte chemoattractant protein-1, macrophage inflammatory protein (MIP)-1α, MIP-1β MIP-2, and vascular endothelial growth factor) and by inhibiting the production and secretion of metabolic factors (soluble glucagon, gastric inhibitory polypeptide, glucagon-like peptide-1, amylin, insulin, leptin, pancreatic polypeptide, and resistin). The authors also demonstrated that MK2-knockout macrophages produced decreased levels of pro-inflammatory IL-10 and metabolic factors and increased levels of IL-12, and exhibited enhanced tumor cytotoxicity, both in vitro in RipTag2 pancreatic cancer cells derived from transgenic mice and in vivo [12].

Cancer cell survival is methionine-dependent, and methionine gamma-lyase (MGL), derived from different non-human organisms, is effective in depleting methionine. Bondarev et al. evaluated the anti-tumoral activity in different types of cancer cell (breast, lung, colon, and neuroblastoma) of a genetically engineered MGL fusion protein (MGL S3), which was formed by fusing MGL from *Clostridium sporogenesis* to epidermal growth factor (EGF)-like peptide. Thus, MGL S3 is selective for EGFR-positive cancer cells. The authors demonstrated that the treatment of cells with MGL S3 decreased cancer cell numbers, downregulated ERK activity, and induced apoptosis in the SH-SY5Y, SK-BR-3, H1299, and HCT116 cell lines. Further, they demonstrated that MGL S3 treatment increased the sensitivity of H1299 cancer cells to the EGFR inhibitor gefitinib, suggesting a novel combined anticancer strategy [13].

Breast cancer-associated fibroblasts (BCAFs) are present in the breast tumor microenvironment, and strongly promote the growth of breast cancer cells through paracrine interactions. Romano et al. demonstrated the anti-tumoral effects of deactivated BCAFs in breast cancer (reverted BCAFs). They generated and characterized the activation, inflammatory, and mesenchymal status of BCAFs grown as monolayers, as spheroids, or as reverted BCAFs obtained from BCAF spheroids reverted to 2D cell adhesion growth. The reverted BCAFs showed a deactivated and anti-inflammatory status, displaying decreased expression of α-smooth muscle actin and cyclooxygenase 2 proteins, and their condition medium exerted a cytostatic effect on MCF-7 cells. Additionally, the conditioned medium from the reverted BCAFs inhibited the migration of MCF-7 cells, suggesting that reverted BCAFs also exhibited decreased pro-tumoral activity. Thus, this study reported the development of new therapeutic strategies that target BCAFs and their interactions with cancer cells [14].

Ratajczak-Wielgomas et al. evaluated the effect of the expression of epithelial periostin (POSTN) in non-small-cell lung cancer (NSCLC) migration and invasion. POSTN is an extracellular matrix N-glycoprotein that has key role in the regulation of cell behavior and the organization of the ECM. POSTN was found to be more highly expressed in the cancer cells of NSCLC patients compared to adjacent non-malignant lung tissue samples. The authors found a significant association of POSTN expression in cancer cells with MMP-2 expression and with clinical stages. Further, POSTN silencing using shRNA (short-hairpin RNA) significantly inhibited A549 lung cancer cells’ migratory and invasive properties by blocking the αvβ3 integrin/PI3K/AKT signaling pathway and by decreasing MMP-2 protein levels. In light of these results, the authors hypothesized that periostin would be a poor prognostic indicator of NSCLC and represents a potential therapeutic target [15].

Finally, the review by Oriuchi et al. discussed the role of 2-deoxy-2-[18F] fluoro-D-glucose (FDG) positron emission tomography (PET) in evaluating the effectiveness of cancer therapy using immune-checkpoint inhibitors, considering that the reactivation of immune cells, which show increased glucose metabolism, might indicate apparent tumor progression on morphological imaging. Accordingly, the authors indicated the need to establish effective biomarkers for monitoring therapy, considering the variability in patients’ therapeutic responses and tumor heterogeneity. With regard to improving the accuracy and reliability of response evaluation, the authors highlighted the benefits of using a radiomics approach that merges objective image features with a machine learning algorithm, as well as pathologic and genetic information, according to tumor heterogeneity and individual variation in therapy response [16].
